# Physico-Chemical Characterization of Tunisian Canary Palm (*Phoenix canariensis* Hort. Ex Chabaud) Dates and Evaluation of Their Addition in Biscuits

**DOI:** 10.3390/foods9060695

**Published:** 2020-05-28

**Authors:** Mohamed Turki, Letricia Barbosa-Pereira, Marta Bertolino, Ismahen Essaidi, Daniela Ghirardello, Luisa Torri, Nabiha Bouzouita, Giuseppe Zeppa

**Affiliations:** 1 Innovation and valorisation laboratory for a sustainable food industry, Higher School of Food Industries, Tunis 1003, Tunisia; mohamedturki231@gmail.com (M.T.); saidi.ismahen@gmail.com (I.E.); bouzouita.nabiha@gmail.com (N.B.); 2 Department of Agriculture, Forest and Food Sciences (DISAFA), University of Turin, 10095 Grugliasco, Italy; letricia.barbosa.pereira@usc.es (L.B.-P.); marta.bertolino@unito.it (M.B.); daniela.ghirardello@unito.it (D.G.); 3 Department of Analytical Chemistry, Nutrition and Food Science, Faculty of Pharmacy, University of Santiago de Compostela, 15705 Santiago de Compostela, Spain; 4 Research Unit of Agrobiodiversity, Higher Agronomic Institute of Chott Meriem, Sousse University, Chott Mériem 4042, Tunisia; 5 University of Gastronomic Sciences, 12042 Bra, Italy; l.torri@unisg.it

**Keywords:** *Phoenix canariensis*, date, biscuit, polyphenol, antioxidant capacity, palm

## Abstract

*Phoenix canariensis* Hort. Ex Chabaud, also known as the Canary Island palm or ornamental palm, is an endemic species of the Canary Islands and has been widely propagated globally. It has become one of the most important and appreciated ornamental plants, especially in the Mediterranean climate. The fruits are edible but used only for feed as they are bitter. Despite its diffusion, not much data on the composition of these fruits and their application as food are available. The aim of this study was to define the chemical characteristics, especially those of the polyphenolic constituents, of red and yellow varieties of Canary palm dates, and to evaluate their use alone or in different mixes in biscuit production. The yellow variety had higher quantities of fiber (36.88% DW (Dry Weight)) and polyphenolic compounds, while the red variety had a high content of sugars, mainly glucose (22.8% DW). Epicatechin is the most important polyphenol of dates (562 μg/g DW). The use of date palm powder on biscuit production resulted in an increase in hardness, polyphenol and fiber content, and antioxidant activity. Sensory analysis showed that the biscuits obtained with a 25/75 mix of red/yellow date powder had the most overall liking.

## 1. Introduction

*Phoenix canariensis* Hort. Ex Chabaud, also known as the Canary Island palm or ornamental palm, is a species that belongs to the Arecaceae family. It is endemic to the Canary Islands, has been widely propagated all over the world, and has become one of the most important and appreciated ornamental plants, especially in the Mediterranean climate [1].

The fruit, which ripens in early summer, is an oval, reddish to dark purple drupe measuring about 2 cm long, with a diameter of 1 cm [2,3] and contains a single large seed, nearly 1 cm long. Sugar concentration varies from 9% to 29% of fresh fruit, according to the maturation stage (from Khalal or yellow stage to Rutab or soft ripe stage) [4]. Moreover, malic acid (0.6%–1% of fresh fruit), citric acid (0.7%–0.8% of fresh fruit), and succinic acid (0.6%–1.8% of fresh fruit) are also present [4]. The polyphenol content is very high, at 85–180 mg of gallic acid equivalent (GAE)/ 100 g of fresh fruit, according to Amorós et al. [4] or 315–2600 mg GAE/100 g of fresh fruit, according to Djouab et al. [5]. The antioxidant capacity is approximately 94% for the peel and 58% for the pulp [4].

The seed is characterised by a high content of oil (about 10%) with approximately 50% of oleic acid and 19% of linoleic acid [6,7].

Although the fruit is edible [4,7], it is very astringent, due to a high polyphenol content [5], not suitable for human consumption, and therefore is used only as feed. Small quantities of fruits have been reported to be consumed locally in the Canary Islands [4], but the only application was as an antioxidant in margarine production [8] as a peel extract. Since *P. canariensis* is extensively planted and produces a large amount of fruit, there is a great amount of available biomass that can be valorized directly or by recovering its bioactive molecules for use as ingredients in food, pharmaceuticals, and cosmetics. The objectives of this study were to perform a physico-chemical characterization of two local Tunisian varieties (red and yellow) of *P. canariensis*, to define their polyphenolic constituents as a first-reported study, and to evaluate their effect alone or as a mix in biscuit production in order to develop a new food application for these fruits.

## 2. Materials and Methods 

### 2.1. Chemicals

Methanol (≥99.9%), formic acid (98%–100%), 6-hydroxy-2,5,7,8-tetramethylchroman-2-carboxylic acid (97%; Trolox), Folin–Ciocalteu’s phenol reagent (2 M), sodium carbonate (≥99.5%), 2,2-diphenyl-1-picrylhydrazyl (DPPH), rutin hydrate (≥94%), (+)–catechin hydrate (>98%), and hexane (≥97.0%) were of analytical grade and were purchased from Sigma-Aldrich, Co (Milano, Italy). Ethanol (≥99.9%), gallic acid (3,4,5-trihydroxy-benzoic) (≥98%), epicatechin (≥99%), quercetin-3-*O*-glucoside (≥98%), *p*-coumaric (>98%), syringic acid (>98%), and caffeic acid (≥95%) were obtained from Fluka (Milano, Italy). *o*-Coumaric and *m*-coumaric acids (≥90%) were obtained from Extrasynthese (Genay, France). Ultrapure water was prepared in a Milli-Q filter system (Millipore, Milan, Italy).

### 2.2. Fruit Samples

Red and yellow varieties of *P. canariensis* dates were bought from the "Nizar Jlassi, Aménagement espaces verts" company from the region of Borj El Amri (30 km southwest of Tunis, Tunisia). The fruits were washed, manually pitted, dried in an oven at 40 °C with forced air until 5% of moisture was reached, and then ground using a ZM200 grinder (Retsch Gmbh, Haan, Germany). The powders were sieved and the fraction between 200–250 µm was selected. The powders were stored in vacuum-sealed polyethylene bags at 4 °C until analysis.

### 2.3. Biscuit Preparation

Biscuits were produced according to the AACC Method 10-53.01 [9] using sugar (100 g), salt (1 g), baking powder (7 g), shortening agent (90 g), water, wheat flour, and date powder. 

Considering a water content for a baked biscuits of about 5% (https://www.alimentinutrizione.it/sezioni/tabelle-nutrizionali), a preliminary test was done where a lot of biscuits were produced with an aliquot of wheat flour substituted with red and yellow date powders in purity to obtain final products with 5%, 7%, 9%, and 11% of date powder. A consumer test was performed with 17 consumers (data not shown) and obtained results highlighting that the maximum percentage acceptable for red and yellow date powder was 9% and 7%, respectively.

According to these results, six types of biscuits with different quantities of red and yellow date powder were produced (Table 1). All productions were done in duplicate.

### 2.4. Physico-Chemical Analysis of Fresh Dates

The dry matter content of fresh dates was determined at 105°C using a Gibertini Eurotherm electronic moisture balance (Gibertini Elettronica, Novate Milanese MI, Italy) using 5 g of fruit.

Ash was obtained after mineralization of the samples in a muffle furnace at 550 °C for 6 h [10]. Protein content was calculated multiplying the nitrogen content evaluated with the Kjeldahl method by 6.25. Fat content was measured by using a Soxhlet extraction apparatus with petroleum ether as a solvent for 6 h [10]. 

Total, insoluble, and soluble fiber content were determined according to the AOAC 991.43 [11].

Sugars and organic acids were determined with liquid chromatography, according to the method described by Bertolino et al. (2011) [12], with slight modifications.

Fresh date (1 g) was added to 10 mL of ultra-pure water, treated for 10 min with an ultrasonic bath, and then centrifuged for 10 min at 10,000× *g* at 10 °C. The supernatant was filtered through a 0.45-µm polypropylene membrane filter and stored at −18 °C until analysis.

The HPLC system (Thermo Quest, San Jose, CA, USA) was equipped with an isocratic pump (P4000), a multiple autosampler (AS3000) fitted with a 20 µL loop, a UV detector (UV100) set to 210 nm and 290 nm, and a refractive index detector RI-150. Data were collected using ChromQuest ver. 3.0 (Thermo Finningan, San Josè, CA, USA).

The analyses were performed isocratically at 0.8 mL/min and 65 °C with a 300× 7.8 mm i.d. cation exchange column (Aminex HPX-87H) equipped with a cation H^+^ microguard cartridge (Bio-Rad Laboratories, Hercules, CA, USA). The mobile phase was 0.013 N H_2_SO_4_. Identification was achieved by comparison with retention times of authentic standards. 

Color analysis was conducted in transmittance mode on a CM-5 spectrophotometer (Konica Minolta, Tokyo, Japan). L*, a*, and b* CIELab parameters were used to measure the color, where L* is the coefficient of lightness ranging from 0 (black) to 100 (white), a* indicates the colors red–purple (when positive a*) and bluish-green (when negative a*), and b* denotes the colors yellow (when positive b*) and blue (negative b*). 

All analyses were performed in triplicate. 

### 2.5. Physico-Chemical Analysis of Biscuits

The water activity was determined at 25 ± 0.02 °C using an AquaLab Pre water activity meter Aqua-Lab CX-2T (Decagon Devices, Pullman, WA, USA). The loss of water during baking was calculated as the difference, for six biscuits, between the weight before and after baking and expressed as a percentage. Total, insoluble, and soluble fiber contents were determined according to the AOAC 991.43 [11]. Color evaluation was performed on crushed biscuits, as described for the date samples. 

The spread factor was calculated according to the AACC Method 10-52.02 [13] as the ratio between the width (W) and the thickness (T) of biscuits multiplied by the correction factor.

Textural analyses of dough and biscuits were performed by the TA.XT2i Plus Texture Analyzer® (Stable Micro System, Godalming, UK) equipped with a 50-kg and a 25-kg load cell, respectively. For the acquisition of the force–time curve, Texture Expert Exceed software 2.54 (Stable Micro System, Godalming, UK) was used. For each matrix, six samples were analyzed.

Dough hardness (N), cohesiveness (adimensional), adhesiveness (mJ), gumminess (N), springiness (mm), and resilience (adimensional) parameters were evaluated using an SMS P/100 probe (Stable Micro System, Godalming, UK). The texture analyzer setting was as follows: pre-test speed of 1 mm/s, test speed of 1 mm/s, post-test speed of 1 mm/s, a distance of 60%, and a trigger force of 5 g.

Biscuit hardness (N) and the area of cutting strength (N/mm) were measured using an HDP/BS probe (Stable Micro System, Godalming, UK). The texture analyzer setting was as follows: pre-test speed of 10 mm/s, test speed of 1 mm/s, post-test speed of 10 mm/s, a distance of 18 mm, and a trigger force of 5 g.

### 2.6. Extractions of Polyphenols 

The extraction of polyphenols was performed according to the method described by Alahyane et al. [14], with slight modifications. Briefly, 1 g of the date powder was mixed with 30 mL of ethanol/water solution (80/20, *v*/*v*), and the extraction was performed at 25 °C for 2 h with a VDRL 711 orbital shaker (Asal S.r.l., Milan, Italy) under constant rotatory agitation at 60 rpm. All extracts were centrifuged at 2800× *g* for 10 min at 4 °C, and the supernatants were then collected and filtered through a 0.45-µm nylon membrane filter. The samples were stored in amber vials at −18 °C. All extractions were done in triplicate.

For biscuit samples, fat was removed with hexane using a solid–liquid extraction protocol. Briefly, 1 g of the biscuit was mixed with 5 mL hexane using a ZX3 advanced vortex mixer (Velp Scientifica, Milan, Italy) for 5 min. All samples were centrifuged at 2800× *g* for 10 min at 4 °C. These operations were repeated two times. Thereafter, hexane was evaporated with nitrogen, and ethanolic extractions were performed as previously described for the fruit samples.

### 2.7. Total Phenolic Content

The total phenolic content (TPC) of extract was determined according to the Folin–Ciocalteu colorimetric method adapted to a 96-well microplate [15] using a BioTek Synergy HT spectrophotometric multi-detection microplate reader (BioTek Instruments, Milan, Italy). All determinations were performed in triplicate. A calibration curve of gallic acid (20–100 mg/L) was constructed to quantify the concentration, which was expressed in milligrams of gallic acid equivalents per gram of dry powder (mg GAE/g DW).

### 2.8. Antioxidant Capacity 

The antioxidant capacity of the extracts was determined by the 2,2-diphenyl-1-picrylhydrazyl radical (DPPH^*^) radical scavenging method described by Barbosa-Pereira et al. [15].

All the assays were conducted in triplicate in 96-well microplates with the by BioTek Synergy HT spectrophotometric multi-detection microplate reader (BioTek Instruments, Milan, Italy). Antioxidant capacity was calculated as the inhibition percentage (IP) of DPPH radical as follows:

(1) IP (%) = ((A_0_ − A_30_)/A_0_) × 100

where A_0_ is the absorbance of the blank and A_30_ is the absorbance at 30 min.

A standard curve of Trolox was constructed (12.5–300 μM) for the assessment of the radical-scavenging activity (RSA) values, which were expressed as micromoles of Trolox equivalent per gram of dry powder (μmol TE/g).

### 2.9. Synergistic Effect

The synergistic effect (SE) of date powder was evaluated on biscuits using the RSA values. Then the SE was used to highlight if the RSA of two powders mixed was higher than the sum of the RSA value of each powder alone [5]. SE was calculated as follows:

(2) SE = RSAe/RSAc

where RSAe is the RSA value determined for the yellow and red mixed powder, whereas the RSAc is the RSA value calculated as follows:

(3) RSAc = a1 × RSAY + a2 × RSAR

where RSAY and RSAR are the RSA values of yellow and red powders, respectively, and a1 and a2 are the quantities of powders in the mix. If SE > 1, a positive synergistic effect between the two powders is indicated.

### 2.10. RP-HPLC–PDA Analysis of Polyphenols

Chromatographic analysis of polyphenols was performed with an HPLC-PDA Thermo-Finnigan Spectra System (Thermo-Finnigan, Waltham, MA, USA). The system was equipped with a P2000 binary gradient pump, an SCM 1000 degasser, an AS 3000 automatic injector, and a Finnigan Surveyor PDA Plus detector. ChromQuest software (Thermo-Finnigan, Waltham, MA, USA, version 5.0) was used for instrument control as well as data collection and processing.

Before chromatographic analysis, the phenolic extracts were purified in Discovery DPA-6S columns (Supelco, Bellefonte, PA, USA). After their activation with 5 mL of methanol, the columns were conditioned with 5 mL of ultra-pure water. After charging the different pre-diluted phenolic extracts with an ethanol/water solution (2:8 *w*/*w*), columns were washed with 5 mL of ultra-pure water to remove the sugars. Samples were eluted first with 5 mL of acetone/formic acid (0.1%) (7:3 *w*/*w*) and then with 5 mL of pure acetone. The last evaporation was done using a nitrogen evaporator (Glas-Col, Terre Haute, IN, USA) at a temperature of 35 °C. All phenolic extracts were reconstituted in ethanol/water (8:2 *w*/*w*) and then filtrated through a 0.2-μm GHP Acrodisc 13-mm syringe filter (Pall, Buccinasco, Italy).

The compounds were separated on a reverse phase Kinetex Phenyl-Hexyl C18 column (150 × 4.6 mm internal diameter and 5-μm particle size) (Phenomenex, Castel Maggiore, Italy) thermostated at 35 °C. A gradient elution method was applied. The following solvents constituted the mobile phase: 0.1% formic acid (solvent A) and 100% methanol (solvent B). The elution conditions were as follows: 0 min–2 min, 90% A and 10% B; 2 min–18 min, linear gradient from 10% to 50% B; 18 min–40 min, 50%–80% B; 40 min–42.0 min, 80%–90% of B; and 42 min–45.0 min, 90%–10% of B; 45.0 min 90% A and 10% of B. The mobile phase flow rate was 1.0 mL/min, and the sample injection volume was 10 μL. Scanning was performed continuously at wavelengths between 200 nm and 500 nm, and data were acquired at 325 nm for *o*-coumaric, *m*-coumaric, *p*-coumaric, and caffeic acids; 279 nm for epicatechin and catechin; 270 nm for gallic acid; and 355 nm for rutin, quercetin-3-*O*-glucoside, and quercetin-3-*O*-glucoside derivates 1 and 2. Quantification was assessed using the external linear calibration curves determined under the same conditions with correlation coefficients > 0.99 (Supplementary Materials Table S1).

### 2.11. Liking Test

The sensory test was conducted with 115 adult subjects (females = 80%; age range: 18–58 years), who were recruited among the staff and students of the University of Gastronomic Sciences and the University of Turin. The study was approved by the Ethics Committee of the University of Gastronomic Sciences. Written informed consent was collected from participants before the test. Participants received individual trays with six biscuit samples and rinsed their mouths with noncarbonated water before beginning the evaluation. Participants tasted the samples according to the tray presentation order, blind, without any information about the innovativeness of the biscuits, to avoid a potential effect of the information on liking scores. Participants rated their liking for appearance, odor, taste, flavor, texture, and overall liking using a 9-point hedonic scale (1 = extremely dislike, 9 = extremely like [16]). Purchase interest (will you buy this biscuit?) was also rated on a 7-point scale (1 = absolutely no, 7 = absolutely yes). Biscuit prototypes were served in a randomized and balanced order. Participants were required to rinse their mouths with still water for about 1 min between samples. Consumers took 10–15 min to complete the evaluation.

### 2.12. Statistical Analysis

Physico-chemical data were subjected to a one-way analysis of variance (ANOVA) with Duncan’s post hoc test at a 95% confidence level, while the Kruskal–Wallis test was used for the statistical analysis of the liking test results.

Data analyses were performed using Statistica 13.3 software (StatSoft, Inc., Tulsa, OK, USA).

## 3. Results and Discussion

### 3.1. Physico-Chemical Characteristics of Fresh Dates

The water content of the yellow *P. canariensis* date variety was 68.55% and that for the red variety was 58.01% (Table 2). These values were significantly different (*p* < 0.05) and this difference may be due to the time of harvest, amount of rainfall, and the nature of the soil. These values were higher than those reported for *Phoenix dactylifera* L. “Deglet Nour” (37.84%–58.44%) by Al-Asmari et al. [17] and *P. dactylifera* L. “Siwi” by Di Cagno et al. [18], which had a water content of 39.9%. However, the values observed were similar to the moisture values of *P. dactylifera* L. reported by Baliga et al. [19], which ranged from 50% to 80% at the Khalal stage (also known as the color stage).

The ash content ranged from 2.11% to 8.98% dry weight (DW) for red and yellow varieties, respectively, and this may be due to the characteristics of the soil and presence of mineral salts [20].

These results were higher than those reported by Chaira et al. [21] for the two cultivars of *P. dactylifera* L., “Deglet-Nour” and “Allig”, which displayed ash contents of 1.30% and 1.11% DW, respectively. Nevertheless, the ash content of red *P. canariensis* was comparable to the values determined by Kchaou et al. [22] for six-second grade cultivars of Tunisian dates that ranged from 2.11% to 3.06% DW.

Sucrose was present only in the red variety, and glucose and fructose concentrations were higher in red *P. canariensis* compared to the yellow variety. The total sugar content varied between 27.8% DW for yellow fruits to 56.7% DW for the red variety. These values were lower than those found by Bouhlali et al. [20] for eight Moroccan date varieties (66.03%–83.05% DW) and those reported by Elleuch et al. [23] for date by-products (72.8%–79.1% DW). However, the sugar content of the yellow date was close to that determined by Amorós et al. [4] for Spanish *P. canariensis* in the Khalal stage (4.07% for glucose; 5.27% for fructose; 0.06% for sucrose, and 9.4% of fresh matter for total sugar).

Contents of malic acid and citric acid were significantly higher in the yellow variety of *P. canariensis*. For Spanish *P. canariensis* fruits, Amorós et al. [4] reported a concentration of 6.4–11.1 mg/g of fresh matter for malic acid and a concentration of 7.2–8.2 mg/g of fresh matter for citric acid, while for 21 Emirati date varieties, Ghnimi et al. [24] reported a concentration from 0.86 to 3.43 mg/g of fresh matter for malic acid and a concentration from 0.11 to 1.00 mg/g of fresh matter for citric acid.

Dates have a low protein content [25], and in this study, the protein content ranged from 1.07% to 1.19% DW and the red variety contained a significantly higher amount. These values were lower than those reported by Kchaou et al. [22] for six varieties of Tunisian dates, which ranged from 2.07% to 3.87% DW but were similar to those reported by Baliga et al. [19], with protein content ranging from 1.10% to 2.60% DW for the varieties of *P. dactylifera* L.

Dates also have a low fat content [25] and in our study, the lipid content ranged from 0.33% to 0.78% DW. The highest values were recorded for the yellow varieties that showed a lipid content higher than those reported by Bouhlali et al. [20] for eight Moroccan date varieties, where the lipid content ranged from 0.218% to 0.363% DW but lower than those observed by Kchaou et al. [22] for six Tunisian varieties of *P. dactylifera* L., where the lipid content ranged from 0.97% to 3.81% DW.

The fiber (soluble, insoluble, and total) content of the yellow variety of *P. canariensis* was more than 2-fold greater than that of the red variety. The total fiber content of yellow variety was higher than that of date fruits of *P. dactylifera* (6.5%–11.5% of fresh fruit) reported by Ghnimi et al. [25].

The brightness values (L*) of the two date varieties varied between 35.02 for the red and 47.71 for the yellow fruits, whereas the a* parameter (redness) varied from 13.87 for the yellow fruits to 24.88 for the red fruits. These significant differences between the two varieties were probably due to the presence of carotenoids, similar to other fruits [4,26,27,28].

Djouab et al. [5] determined the color parameters of Algerian *P. canariensis* red date specimens and found values of 31.80 ± 0.85 for L*, 37.80 ± 1.53 for a*, and 10.20 ± 0.46 for b*. Amorós et al. [4] determined the color parameters for Spanish *P. canariensis* yellow dates and the obtained values (L*=65.15, a*=22.50, and b*=47.80) were in accordance with the results found in our study.

The total phenolic content (TPC) and the free radical scavenging activity (RSA) of the *P. canariensis* date powders are shown in Table 3, where values for the mixes used for biscuit production are also shown. The yellow date showed the highest values of TPC and RSA, which were approximately 9-fold higher than those observed for the red date.

Djouab et al. [5] reported a TPC of 26.00 ± 1.10 GAE/g DW for the Algerian red Canary date, which was similar to that observed in this study, while Amorós et al. [4] reported a TPC of 1.79 ± 0.03 GAE/g fresh matter for the Spanish yellow Canary date.

This difference could be due to differences in extraction method but also to differences in culturing technique and maturity of the fruit, as observed by other authors for *P. dactylifera* dates [29,30,31].

The values of the synergistic effects (SEs) evaluated for the mixture of yellow and red variety powders were always higher than 1, highlighting a positive interaction between the date powders. In particular, the SE values were 1.30 for 25/75 R/Y, 1.05 for 50/50 R/Y, and 1.07 for 75/25 R/Y. Allane and Benamara [32] found an SE value between 1.29 and 2.24 for four combinations of peels of three fruits (arbutus/garnet wild stone date/black grapes).

Eleven polyphenolic compounds were identified and quantified in this study on dried *P. canariensis* dates: caffeic acid, gallic acid, *m*-coumaric acid, *p*-coumaric acid, *o*-coumaric acid, catechin, epicatechin, rutin, quercetin-3-*O*-glucoside, and the two derivates of quercetin-3-*O*-glucoside (Table 4). In yellow dates, gallic acid, *m*-coumaric acid, catechin, epicatechin, rutin, quercetin-3-*O*-glucoside, and the two derivates of quercetin-3-*O*-glucoside were detected, while for the red variety all phenolic compounds were detected except the quercetin-3-*O*-glucoside derivate 1. Ghnimi et al. [25] reviewed and reported the phenolic compounds identified in the *P. dactylifera* date fruits. All varieties contained mainly catechin, epicatechin, rutin, quercetin-3-*O*-glucoside, gallic acid, and some cinnamic acid derivatives.

Epicatechin, gallic acid, and quercetin-3-*O*-glucoside derivate 2 were the most important polyphenolic compounds in both *P. canariensis* dates with significant differences between the varieties. In particular, the amounts of phenolic compounds in the yellow variety were higher than those in the red variety, with the exception of *m*-coumaric acid. The epicatechin content in the yellow variety was approximately five times higher than that in the red variety. These values were also higher than those determined by Sheikh et al. [33] (91.5–219.3 µg/g of dry extract) for three Saudi Arabian *P. dactylifera* date varieties. The gallic acid contents of the *P. canariensis* dates studied here, especially for the yellow variety, were higher than those determined by Alahyane et al. [14] (43.7–314.11 µg/g DW), Bouhlali et al. [30] (55.3–103.8 µg/g DW), and Al Harthi et al. [34] (70–191.4 µg/g fresh matter) for different varieties of *P. dactylifera* fruits. For the yellow variety, the catechin content was in the same range of the values as determined by Alahyane et al. [14] (18.67–72.75 µg/g DW) in *P. dactylifera* fruits, while quercetin-3-*O*-glucoside content in the yellow fruit was higher than that determined by Khallouki et al. [31] (19.05 µg/g of fresh matter) for mature *P. dactylifera* Moroccan Medjool fruits. Moreover, compared to the values determined by Benmeddour et al. [35] (1.6–30.3 µg/g DW) for ten cultivars of Algerian *P. dactylifera* fruits, the two *P. canariensis* fruits showed higher quercetin-3-*O*-glucoside contents. The caffeic acid content of the red variety was similar to the values found by Alahyane et al. [14] (3.61–8.35 µg/g DW) for *P. dactylifera* fruits and higher than that determined by Benmeddour et al. [35] (0.3 µg/g–1.2 µg/g DW) for ten Algerian *P. dactylifera* date cultivars. The rutin content of the yellow variety was higher than that determined by Bouhlali et al. [30] (5.2–28.6 µg/g DW) and Benmeddour et al. [35] (1.7–27.8 µg/g DW) for *P. dactylifera* fruits, while the rutin content of the red fruits was higher than that determined by Alahyane et al. [14] (7.38–20.12 µg/g DW) for 17 Moroccan *P. dactylifera* date varieties and clones. In contrast to the yellow fruits, the red fruits contained higher amounts of *m*-coumaric acid (12.5–25.6 µg/g fresh fruits) than those determined for four common varieties of Tunisian *P. dactylifera* fruits [36]. The *p*-coumaric acid content of red fruits was superior to that determined by Bouhlali et al. [30] (4.9–17.4 µg/g DW) and by Benmeddour et al. [35] (0.9–4.8 µg/g DW) for *P. dactylifera* fruits. The *o*-coumaric acid content of red fruits was present in the range determined by Alahyane et al. [14] (0.57–4.81 µg/g DW) for the Moroccan *P. dactylifera* fruits.

The two varieties of *P. canariensis* fruits contained several compounds that were present in high amounts, such as epicatechin, gallic acid, and the quercetin-3-*O*-glucoside derivate 2, and this could be the reason for the high antioxidant capacity found in these fruits, as highlighted by Pico et al. [37] for banana flour, where its antioxidant capacity was directly correlated to epicatechin, gallic acid, and the quercetin-3-*O*-glucoside content.

### 3.2. Physico-Chemical Characteristics of Biscuits 

The replacement of wheat flour by *P. canariensis* powders produced a change in biscuit humidity and spread (Table 5). Products obtained with date powder had a humidity higher and generally a spread lower than the control. These results are similar to those obtained by Protonotariou et al. [38] and Sudha et al. [39] for biscuits produced with the substitution of whole wheat flour with rice and oat bran blends.

The biscuits obtained with only yellow date powder (100 Y) showed the highest moisture content (6.77%), and this could be due to the richness of *P. canariensis* dates in fiber (especially the yellow varieties). These results were supported by a significant increase of water activity observed for the biscuits with date powder added. Moisture values were higher than those obtained for biscuits fortified with mustard (2.55%–3.15%) and biscuits supplemented with spinach (0.94%–1.26%), described by Tyagi et al. [40] and Rao Galla et al. [41], respectively. However, these values were lower than the ones obtained from pigeon pea–wheat composite flour studied by Gbenga–Fabusiwa et al. [42], which ranged from 9.84% to 12.93%.

The loss of water during baking ranged from 5.39% to 6.55% with a significant difference between samples (*p* < 0.05).

The fiber content ranged from 2.23 to 5.44 g/100 g. As the percentage of *P. canariensis* date powder added increased, the biscuit fiber content increased, more effectively with yellow date powder addition than red. The addition of yellow date powder increased the fiber content of the biscuit.

Texture evaluation was performed on the dough and the baked biscuits. All the doughs obtained after substituting wheat flour with date powder showed significantly (*p* < 0.05) higher hardness than the control dough biscuits obtained with only wheat flour (Table 6). Only the 100 R dough showed a hardness value lower than the control. The lower fiber content of the red date (Table 5) compared to the yellow date may explain this difference, and although hardness generally increased with the fiber content in biscuit doughs, there were some exceptions [41].

The cohesiveness and the adhesiveness of doughs were positively correlated with the hardness (*r* = 0.90 and 0.89, respectively). The 100 R dough was characterised by the lowest insoluble fiber content and showed the highest adhesiveness (−18.43) and lower resilience. This result confirms the strong correlation between resilience and adhesiveness (*r* = 0.90). The 100 Y dough displayed the highest values for all textural parameters, except for adhesiveness, where 100 Y showed a lower value. This may be related to the high insoluble fiber content. Several authors have mentioned that fiber addition has a bad effect on the biscuit dough texture when the insoluble fraction of the fiber is very high [43].

The replacement of wheat flour by *P. canariensis* powder also affected the texture of biscuits. A significant difference between the control biscuit and all enriched biscuits in terms of hardness (*p* > 0.05) was seen. In particular, the 25/75 R/Y biscuit showed lower hardness and the 50/50 R/Y showed higher hardness. With respect to the rupture work, a significant difference was observed between the control biscuit and 50/50 R/Y and 75/25 R/Y biscuits (*p* < 0.05). This could be due to the low soluble fiber content in these biscuits.

The replacement of wheat flour by date powder significantly reduced the lightness (L*) of the biscuits (*p* < 0.001) but also increased the a* and b* parameters. In particular, for a high content of yellow powder, there was an increase of b* due to the color of the date powder. After milling, the red powder lost its characteristic red color due to its fleshy white pulp, while the yellow powder maintained its characteristic yellow color. Therefore, the yellow variety contributed more than the red variety to a color change. There was no significant difference between 25/75 R/Y and 50/50 R/Y (*p* > 0.05).

Total phenolic content and free radical scavenging activity of biscuits were significantly different among the products. As the yellow powder displayed higher values of TPC and RSA than the red powder, the biscuits obtained with this product showed a significant increment of TPC and RSA, proportional to the increment of yellow powder.

The sensory effect of date powder addition on biscuits was evaluated with an overall consumer liking and purchase interest (Table 7). A significant difference was found in liking among samples for all the examined parameters (appearance, odor, taste, flavor, texture, overall liking, and purchase interest).

In particular, the 25/75 R/Y biscuit exhibited the highest score for taste, flavor, texture, overall liking, and purchase interest. These results could be correlated to its lower hardness and a color with high values of a* and b*. This product also showed a high content of fiber and high values for TPC and RSA.

## 4. Conclusions

The present study provided, for the first time, a complete physico-chemical characterization of fruits obtained from red and yellow varieties of Tunisian *P. canariensis*. High content of fiber and polyphenolic compounds, such as gallic acid, epicatechin, and quercetin derivates, and consequently high values of TPC and RSA, were obtained. Addition of powders obtained from these fruits to wheat flour resulted in biscuits with two-fold fiber and four-fold polyphenolic content to that of biscuits obtained from wheat flour alone. Powder addition had no significant effect on biscuit structure and consumer acceptability, and the 25/75 R/Y biscuit exhibited the highest score for taste, flavor, texture, overall liking, and purchase interest. Thus, *P. canariensis* fruits can be used for new innovative and functional foods valorizing a typical by-product.

## Figures and Tables

**Table 1:** Composition (g) of doughs with different quantities of red (R) and yellow (Y) date powders.

Ingredients	100 Y	25/75 R/Y	50/50 R/Y	75/25 R/Y	100 R	Control
Water	25	25	25	25	25	25
Sugar	100	100	100	100	100	100
Salt	1	1	1	1	1	1
Baking powder	7	7	7	7	7	7
Shortening agent	90	90	90	90	90	90
Wheat flour	242	240	237	234	232	277
Red date powder	0	11	23	34	45	0
Yellow date powder	35	26	17	9	0	0

**Table 2:** Physico-chemical characteristics (mean values ± standard error) of the two fresh Tunisian *P. canariensis* dates and results of variance analysis.

Parameter	Yellow	Red	Significance
Moisture (%)	68.55 ± 1.43	58.01 ± 1.06	**
Ash (%)	8.98 ± 0.02	2.11 ± 0.01	***
Proteins (%)	1.07 ± 0.11	1.19 ± 0.04	ns
Lipids (%)	0.78 ± 0.02	0.33 ± 0.00	**
Sugars (%)	27.8 ± 1.0	56.7 ± 2.7	**
Sucrose (%)	nd	11.8 ± 2	n/a
Glucose (%)	12.5 ± 0.5	22.8 ± 0.3	***
Fructose (%)	15.3 ± 0.5	22.1 ± 0.4	**
Malic acid (mg/g)	18.65 ± 1.25	6.89 ± 0.1	*
Citric acid (mg/g)	4.06 ± 0.21	0.77 ± 0.05	**
Total fiber (%)	36.88 ± 3.5	17.37 ± 1.63	*
Insoluble fiber (%)	27.97 ± 2.6	13.40 ± 1.26	*
Soluble fiber (%)	8.91 ± 0.84	3.97 ± 0.37	*
L *	47.71 ± 0.88	35.02 ± 0.48	***
a *	13.87 ± 0.29	24.88 ± 0.41	***
b *	44.35 ± 1.03	26.81 ± 0.62	***

nd: not determined; n/a: not applicable; data expressed as dry weight; ns: not significant; * *p* < 0.05; ** *p* < 0.01; *** *p* < 0.001.

**Table 3:** Total phenolic content (TPC; mg GAE/g dry weight) and radical scavenging activity (RSA; µmol eq. Trolox/g dry weight) of powder of the two Tunisian *P. canariensis* date varieties and mixes used for biscuit production (R-red; Y-yellow; mean ± standard error) and results of variance analysis with Duncan’s test.

Powder	RSA	TPC
Yellow date	1503.42 ± 61.39 ^a^	202.09 ± 6.23 ^a^
Red date	141.08 ± 19.00 ^d^	23.70 ± 2.66 ^d^
25/75 R/Y	1507.29 ± 53.01 ^a^	201.80 ± 10.39 ^a^
50/50 R/Y	820.65 ± 9.59 ^b^	130.14 ± 1.11 ^b^
75/25 R/Y	513.32 ± 46.88 ^c^	90.53 ± 3.07 ^c^
Significance	***	***

*** *p* < 0.001; data in the same column with different letters were significantly different at *p* < 0.05.

**Table 4:** Content (µg/g dry weight; mean ± standard error) of phenolic compounds of the two Tunisian *P. canariensis* varieties and results of variance analysis.

Phenolic Compound	Yellow	Red	Significance
Gallic acid	416.28 ± 12.81	287.57 ± 3.88	*
Catechin	75.49 ± 0.14	36.33 ± 7.30	*
Caffeic acid	nd	7.15 ± 0.03	n/a
Epicatechin	562.99 ± 5.75	105.11 ± 3.95	***
*p*-coumaric acid	nd	20.51 ± 0.21	n/a
m-coumaric acid	10.58 ± 1.53	95.85 ± 0.83	***
*o*-coumaric acid	nd	1.97 ± 0.70	n/a
Rutin	171.37 ± 13.59	25.62 ± 0.72	**
Quercetin-3-*O*-glucoside	315.04 ± 3.34	39.72 ± 0.36	***
Quercetin-3-*O*-glucoside derivate 1	261.93 ± 4.60	nd	n/a
Quercetin-3-*O*-glucoside derivate 2	428.15 ± 3.55	114.99 ± 3.15	***
Polyphenolic sum	2241.80 ± 45.28	734.80 ± 21.11	

nd: not detected; n/a: not applicable; * *p* < 0.05; ** *p* < 0.01; *** *p* < 0.001.

**Table 5:** Mean values (± standard error) of physico-chemical parameters evaluated on the biscuits and results of variance analysis with Duncan’s test (R-red; Y-yellow).

Parameter	100 Y	100 R	25/75 R/Y	50/50 R/Y	75/25 R/Y	Control	Significance
Humidity (%)	6.77 ± 0.04 ^d^	6.22 ± 0.19 ^cd^	6.38 ± 0.30 ^cd^	5.64 ± 0.12 ^b^	6.00 ± 0.21 ^bc^	4.54 ± 0.33 ^a^	***
Spread	7.64 ± 0.07 ^ab^	8.28 ± 0.18 ^c^	7.33 ± 0.09 ^a^	7.95 ± 0.14 ^bc^	7.43 ± 0.08 ^a^	7.91 ± 0.20 ^bc^	***
Water activity	0.50 ± 0.01 ^d^	0.45 ± 0.02 ^abc^	0.47 ± 0.01 ^bcd^	0.44 ± 0.01 ^ab^	0.48 ± 0.01 ^cd^	0.42 ± 0.02 ^a^	***
Loss of water (%)	6.04 ± 0.31 ^bc^	6.55 ± 0.09 ^c^	5.79 ± 0.09 ^ab^	5.39 ± 0.22^a^	6.07 ± 0.15 ^bc^	5.68 ± 0.21 ^ab^	*
Total fiber (%)	5.44 ± 0.51 ^c^	3.26 ± 0.31 ^ab^	4.99 ± 0.47 ^c^	4.29 ± 0.41 ^bc^	3.4 ± 0.32 ^ab^	2.23 ± 0.21 ^a^	**
Insoluble fiber (%)	4 ± 0.37 ^c^	2.42 ± 0.23 ^a^	3.83 ± 0.36 ^c^	3.49 ± 0.33 ^bc^	2.64 ± 0.25 ^ab^	1.91 ± 0.19 ^a^	*
Soluble fiber (%)	1.44 ± 0.13 ^c^	0.84 ± 0.08 ^b^	1.15 ± 0.11 ^c^	0.81 ± 0.08 ^b^	0.77 ± 0.07 ^b^	0.32 ± 0.03 ^a^	**
Hardness (N)	39.07 ± 4.50 ^a^	39.13 ± 2.71 ^a^	27.92 ± 2.35 ^b^	43.73 ± 2.79 ^a^	42.85 ± 3.25 ^a^	37.36 ± 4.01 ^ab^	***
Rupture work (N/mm)	37.49 ± 3.41 ^ab^	33.56 ± 2.79 ^abc^	24.12 ± 2.84 ^c^	40.12 ± 4.05 ^a^	38.33 ± 3.69 ^a^	27.73 ± 2.99 ^bc^	***
L*	56.41 ± 0.22 ^a^	56.67 ± 0.85 ^a^	55.85 ± 0.73 ^a^	55.78 ± 0.63 ^a^	54.91 ± 0.87 ^a^	73.00 ± 1.19 ^b^	***
a*	10.97 ± 0.11 ^e^	7.04 ± 0.17 ^b^	9.26 ± 0.16 ^d^	9.29 ± 0.11 ^d^	8.33 ± 0.41 ^c^	2.46 ± 0.28 ^a^	***
b*	38.45 ± 1.18 ^e^	24.69 ± 0.40 ^b^	32.57 ± 0.55 ^d^	31.69 ± 0.61 ^d^	27.90 ± 0.22 ^c^	22.41 ± 0.66 ^a^	***
RSA (µmol TE/g DW)	7.77 ± 0.20 ^a^	3.55 ± 0.05 ^e^	6.48 ± 0.14 ^b^	5.57 ± 0.11 ^c^	4.94 ± 0.04 ^d^	0.01 ± 0.00 ^f^	***
TPC (mg GAE/g DW)	1.78 ± 0.04 ^a^	1.09 ± 0.02 ^d^	1.59 ± 0.03 ^b^	1.67 ± 0.05 ^b^	1.48 ± 0.01 ^c^	0.44 ± 0.02 ^e^	***

* *p* < 0.05; ** *p* < 0.01; *** *p* < 0.001; data in the same line with different letters were significantly different at *p* < 0.05.

**Table 6:** Mean values (± standard error) of texture parameter evaluated on biscuit doughs produced with different date powders and results of variance analysis with Duncan’s test (R-red; Y-yellow).

Doughs	Hardness	Cohesiveness	Gumminess	Springiness	Resilience	Adhesiveness
100 Y	445.21 ± 6.30 ^e^	0.36 ± 0.01 ^d^	161.37 ± 4.06 ^e^	0.34 ± 0.01 ^d^	0.23 ± 0.01 ^d^	−8.59 ± 1.21 ^c^
100 R	159.62 ± 5.81 ^a^	0.31 ± 0.01 ^b^	48.88 ± 2.11 ^a^	0.32 ± 0.01 ^cd^	0.11 ± 0.01 ^a^	−18.43 ± 1.50 ^a^
25/75 R/Y	386.96 ± 8.45 ^d^	0.35 ± 0.01 ^d^	137.35 ± 5.55 ^d^	0.32 ± 0.01 ^cd^	0.21 ± 0.01 ^d^	−7.37 ± 0.57 ^c^
50/50 R/Y	294.21 ± 2.09 ^c^	0.33 ± 0.01 ^c^	96.98 ± 2.58 ^c^	0.29 ± 0.01 ^bc^	0.17 ± 0.00 ^c^	−11.98 ± 0.83 ^b^
75/25 R/Y	241.90 ± 9.33 ^b^	0.31 ± 0.01 ^bc^	75.88 ± 3.89 ^b^	0.28 ± 0.0 1^b^	0.15 ± 0.00 ^b^	−14.39 ± 0.70 ^b^
Control	172.57 ± 10.36 ^a^	0.27 ± 0.01 ^a^	46.72 ± 4.28 ^a^	0.25 ± 0.01 ^a^	0.12 ± 0.01 ^a^	−13.17 ± 0.66 ^b^
Significance	***	***	***	***	***	***

*** *p* < 0.001; data in the same column with different letters were significantly different at *p* < 0.05.

**Table 7:** Sum of the ranks for each sensory descriptor of the biscuits and results of Kruskall–Wallis test with mean comparison (R-red; Y-yellow).

Biscuit	Appearance	Smell	Taste	Flavor	Texture	Overall Liking	Purchase
100 Y	45,627 ^c^	46,828 ^c^	37,476.5 ^ab^	37,975 ^ab^	31,521 ^a^	37,346 ^a^	37,495 ^ab^
100 R	35,961.5 ^ab^	35,197.5 ^b^	38,308 ^ab^	37,324 ^ab^	31,378.5 ^a^	36,409.5 ^a^	37,524 ^ab^
25/75 R/Y	43,078.5 ^bc^	46,673 ^c^	45,114 ^b^	45,424 ^b^	44,025 ^b^	46,944.5 ^b^	46,160.5 ^b^
50/50 R/Y	43,676 ^bc^	44,340.5 ^c^	39,579 ^ab^	40,539 ^ab^	36,137 ^ab^	39,150 ^ab^	39,552 ^ab^
75/25 R/Y	41,727 ^bc^	41,494.5 ^bc^	43,146.5 ^ab^	45,167 ^b^	42,133.5 ^b^	43,504 ^ab^	43,939.5 ^b^
Control	28,325 ^a^	23,861.5 ^a^	34,771 ^a^	31,966 ^a^	53,200 ^c^	35,041 ^a^	33,724 ^a^
Significance	***	***	**	***	***	***	***

** *p* < 0.01; *** *p* <0.001; sum of the ranks in the same column with different letters were significantly different at *p* < 0.05.

## Supplementary Materials

The following are available online at https://www.mdpi.com/2304-8158/9/6/695/s1, Table S1: Chromatographic parameters of polyphenolic compounds detected on RP-HPLC–PDA in *P. canariensis* date extracts.
