# Supplementary material for: Physico-Chemical Characterization of Tunisian Canary Palm (Phoenix canariensis Hort. Ex Chabaud) Dates and Evaluation of Their Addition in Biscuits
Source: Foods. 2020 May 28;9(6):695. doi: 10.3390/foods9060695 (PMC7353613; doi:10.3390/foods9060695)
Supplement: Supplementary file 1 [file foods-09-00695-s001.pdf]

Table S1. Chromatographic parameters of polyphenolic compounds detected on RP-HPLC–PDA in *P. canariensis* date extracts.

Compound	Rt (min)	λ_{max} (nm)	Linear range (mg/L)	Linear Equation	R^2	LOD (mg/L)	LOQ (mg/L)
Gallic acid	2.17	270	1–200	y= 40117x − 49148	0.9947	0.2	0.5
Catechin	7.23	279	0.5–194	y= 32160x −14603	0.9992	0.2	0.5
Caffeic acid	9.04	325	0.2–20	y= 273358x − 32405	0.9999	0.1	0.2
Epicatechin	10.38	279	1–198	y= 32367x + 14110	0.9994	0.2	1
p-Coumaric acid	12.63	325	1–50	y= 108154x − 28336	0.9981	0.1	0.2
m-Coumaric acid	14.09	325	1–100	y= 16635x + 9784	0.9946	0.2	0.5
o-Coumaric acid	15.62	325	1–200	y= 106639x+38045	0.9997	0.2	0.5
Rutin	16.8	355	1–50	y= 63025x + 36818	0.9996	0.2	0.5
Quercetin-3-*O*-glucoside	17.13	355	0.5–110	y= 87981x − 27157	0.9999	0.1	0.5
Quercetin-3-*O*-glucoside derivate 1	18.29	355	0.5–110	y= 87981x − 27157	0.9999	0.1	0.5
Quercetin-3-*O*-glucoside derivate 2	19.01	355	0.5–110	y= 87981x − 27157	0.9999	0.1	0.5
